# The Regeneration of Urban Blue Spaces: A Public Health Intervention? Reviewing the Evidence

**DOI:** 10.3389/fpubh.2021.782101

**Published:** 2022-01-13

**Authors:** Anna Brückner, Timo Falkenberg, Christine Heinzel, Thomas Kistemann

**Affiliations:** ^1^Center for Development Research, University of Bonn, Bonn, Germany; ^2^Institute for Hygiene and Public Health, University Hospital Bonn, Bonn, Germany; ^3^Department of Geography, University of Bonn, Bonn, Germany

**Keywords:** urban blue spaces, urban regeneration, wellbeing, public health, urban planning

## Abstract

Research in recent years has demonstrated that urban surface waters (“urban blue spaces”) can provide beneficial effects on human health and wellbeing. Despite blue spaces prevailing on urban development agendas across the world, little investigation has been done whether and how the regeneration of such spaces is used as a (community-based) public health intervention. Therefore, a review was conducted to analyze urban blue space regeneration projects in terms of their significance for public health. Results show that the regeneration of urban blue spaces displays a diversity of intervention types and follows certain development trends seen in general urban regeneration: Similarities mainly arise in relation to objectives (multi-dimensional goals with increasing focus on environmental sustainability and economic interests), stakeholders (shift to multi-actor governance with a rise of partnerships and community participation), and funding (prevalence of mixed financial schemes and increasing reliance on external funding sources). Although threefold public health effects have been noted across the projects (i. behavioral changes toward healthier lifestyles, ii. healthier urban environments, iii. health policy changes), results of this review indicate that the potential to use urban blue regeneration as a community-based health intervention has yet to be realized.

## Introduction

In the 21st century, a main challenge for cities will be to unlock their transformative power for ensuring sustainable urbanization (1), while concurrently tackling questions of economic development and social justice (2). The threats posed by global development trends and local driving forces such as climate change and socio-spatial segregation call for a reinvention of cities and promote the vision of an advanced city model: one that is healthy, lively, safe, and sustainable (3). Among the various urban imaginaries, the “Green City” has become subject of global urban regeneration activities, often aided by deindustrialization (4). Although greening interventions are widespread on urban development agendas to reach environmental and social goals, it can actually be observed that cities worldwide are in the process of also becoming *blue*.

Coined under the term “waterfront revitalization”, urban surface waters (“urban blue spaces”) such as coastlines, rivers and lakes, have gained momentum in urban planning over the last decades (5, 6). The various values ascribed to the regeneration of blue spaces include environmental and social aspirations (e.g., improved eco-health, enhanced urban aesthetics and quality of urban life), and increased economic prospects (7). Further, new approaches to climate change adaptation and mitigation have led to increasing implementation of nature-based solutions (NBS) that combine stormwater management with urban development aims (“water-sensitive urban design”); thus creating small-scale blue-green spaces in cities (8).

From a *public health perspective*, urban blue (and green) spaces are important health-enabling landscapes that can mitigate urban health risks such as air and noise pollution and promote healthy behaviors, e.g., physical activity. For “the blue” (9), research in recent years has increasingly revealed the salutogenic qualities of waters and has demonstrated distinct linkages between blue space exposure and physical, social, and mental wellbeing [e.g., (9–11)]. However, whereas urban greening has gained much recognition and existing initiatives have been analyzed to maximize health-related benefits [e.g., (12, 13)], less is known about “urban blueing”, i.e., urban blue space interventions for improved health in cities.

### Study Objectives

The purpose of this literature review is to identify urban regeneration projects that aim for physical improvements through the provision of urban blue spaces in deprived communities^1^. Such “urban blue regeneration schemes” may change the quantity (i.e., the amount or relative distribution) and/or the quality of blue spaces and its surroundings, including (but not limited to): waterside estate action and housing improvements, ecological restorations of waterbodies, and improvements to blue space design. Ultimately, such interventions affect the access and/or accessibility to blue spaces as well as the types of activities conducted herein. Particularly large-scale projects are likely to induce socio-spatial transformations of adjacent areas with mutual effects on the blue space characteristics, e.g., attraction of new businesses or changes in the local population composition.

With regard to the potential of urban blue spaces for the promotion of human health and wellbeing, this review particularly aims to explore the significance of urban blue regeneration as an environmental (community-based) public health intervention. By comparing common and distinctive project features, this study intends to generate practice-oriented knowledge aimed at guiding urban planning and policy, with the overall intention to provide health-enabling blue spaces throughout cities. Three main questions guide this review:

- Why and how are urban blue spaces regenerated?
- Which role does public health play in urban blue regeneration?
- Which recommendations can be drawn for urban planning and policy?

## Methodology

### Search Strategy

Four scientific databases, Bonnus (University Bonn Library Catalog), Pubmed, ScienceDirect, and Web of Science, were searched using different combinations of keywords and phrases related to the topic under study (see Table 1). Additional to the bibliographic search, we screened data resources on architecture and urban development such as project databases and compilations of best practice in public space design (e.g., European Prize for Urban Public Space), reviews, case studies, and reports on urban regeneration and waterfront revitalization. As blue spaces can form part of urban greening interventions and actions on climate change adaptation and mitigation, we considered as well literature from those fields of landscape architecture. Further sources included screening of recent public health research initiatives on urban blue infrastructure [e.g., (15)] and snowballing. All types of urban blue spaces from cities all over the world were included. Given the global intensification of waterfront revitalization since the mid-1980s and the subsequent increasing research recognition, we looked for urban blue regeneration schemes that have been conducted from 1990 onwards. The search was limited to publications written in English, French, and German. The last search was conducted on 15 March 2020.

**Table 1:** Keyword combinations (in title/abstract) for database searching.

**Blue space**	**Urban regeneration**	**Health**
“Blue space” OR “water*” OR “river*” AND	“Urban” AND “regeneration” OR “renewal” OR “revitalization” OR “rehabilitation” OR “development” OR “redevelopment” OR “reconstruction” OR “slum clearance” OR “upgrading” OR “depriv*” OR “distressed” AND	“Health” OR “wellbeing”

### Selection of Projects: Eligibility Criteria

In light of the wide range of issues that fall in the scope of urban regeneration, we considered all possibly guises:

- Comprehensive, usually long-term and integrated visions and actions that seek to resolve several kinds of urban problems and that are (ideally) designed and carried out in a total set of measures to the final point of completion, and
- Single urban regeneration schemes tackling one particular urban problem that has been defined to be the gist of the matter (mostly seen in practice) (2, 14).

We considered all scales of action, from neighborhood- and area-based initiatives, to metropolitan regional approaches. Only blue space interventions that have been located in deprived communities, either according to the project description or based on available secondary data (e.g., according to the city deprivation index) were included in the review. We included gentrifying areas as well as those that gentrified in the past, i.e., where gentrification processes have taken place after project implementation.

**Figure 1:**
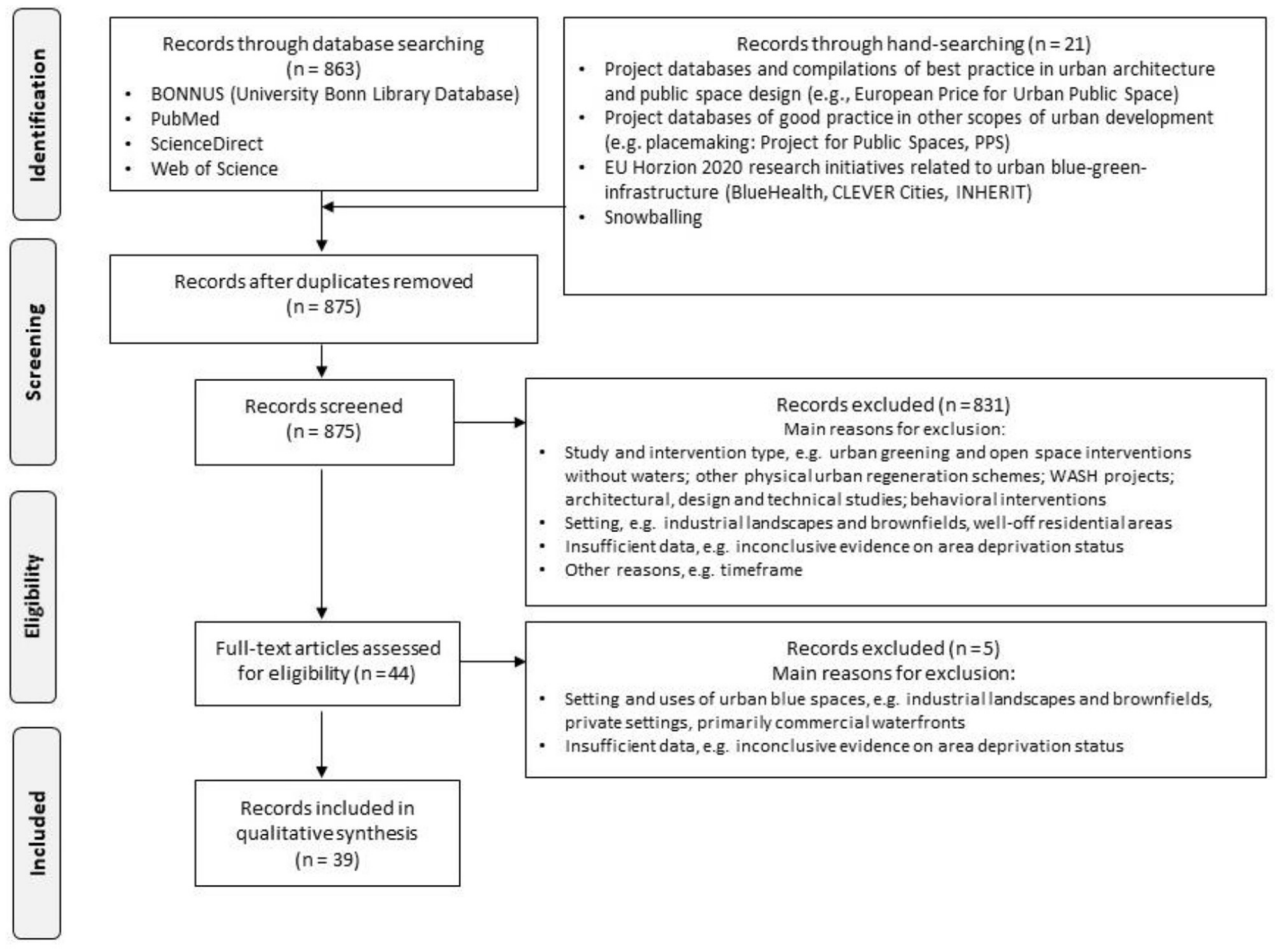
Selection of review projects following the PRISMA flow chart according to (16).

We only included urban regeneration actions of publicly accessible blue spaces in and nearby deprived residential areas or those that created access to blue spaces for deprived communities as a project result. This includes projects in urban areas with mixed land-use if targeting disadvantaged populations explicitly. The focus on residential areas and public accessible blue spaces was chosen to allow comparability between the projects. Conversely, we excluded regeneration schemes in primarily commercial or industrial areas such as the redevelopment of ports. We further excluded projects in private (i.e., owner-occupied) and semi-public settings, e.g., schools. Urban greening initiatives have been included if blue spaces were an integral part of the project. We further excluded behavioral interventions such as swimming or walking groups and projects that exclusively target improved access to water, sanitation and hygiene (WASH). Figure 1 summarizes the stepwise selection process adopted in this review.

### Data Analysis

**Figure 2:**
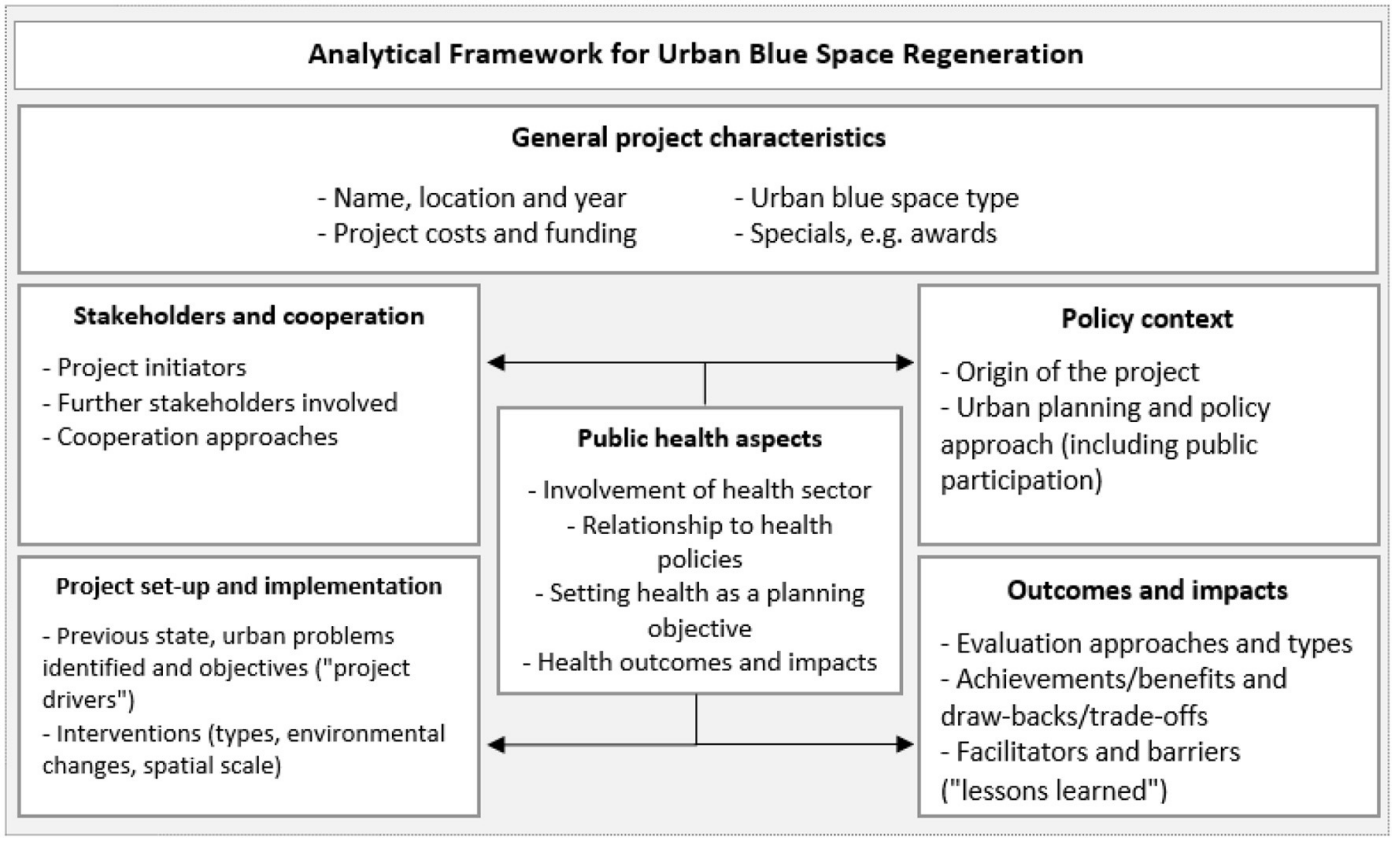
Framework for analyzing urban blue regeneration schemes [modified from (14)].

We created an excel database with all eligible results (*n* = 39) for thematic and content analysis. The database was adapted throughout the data collection and analysis process, incorporating new evaluation criteria as appropriate. To guide the overall analysis, we built upon and modified existing frameworks for urban policy analysis, most notably the “Framework for analyzing urban regeneration: Twelve key questions” by Hall and Barrett (14), and on analytical approaches applied in similar reviews on blue and green space interventions [e.g., (13, 17)]. Finally, the projects are described in terms of parameters on five levels of analysis, as presented in Figure 2. Public health aspects are examined across the different levels, except the general project characteristics.

## Results and Discussion

The following section first presents and interprets the key findings of each level of analysis in the context of literature, second, discusses the role of public health in urban blue regeneration, and third, draws policy recommendations.

### General Project Characteristics

The general project characteristics are summarized in Table 6 in the Supplementary Material.

**Figure 3:**
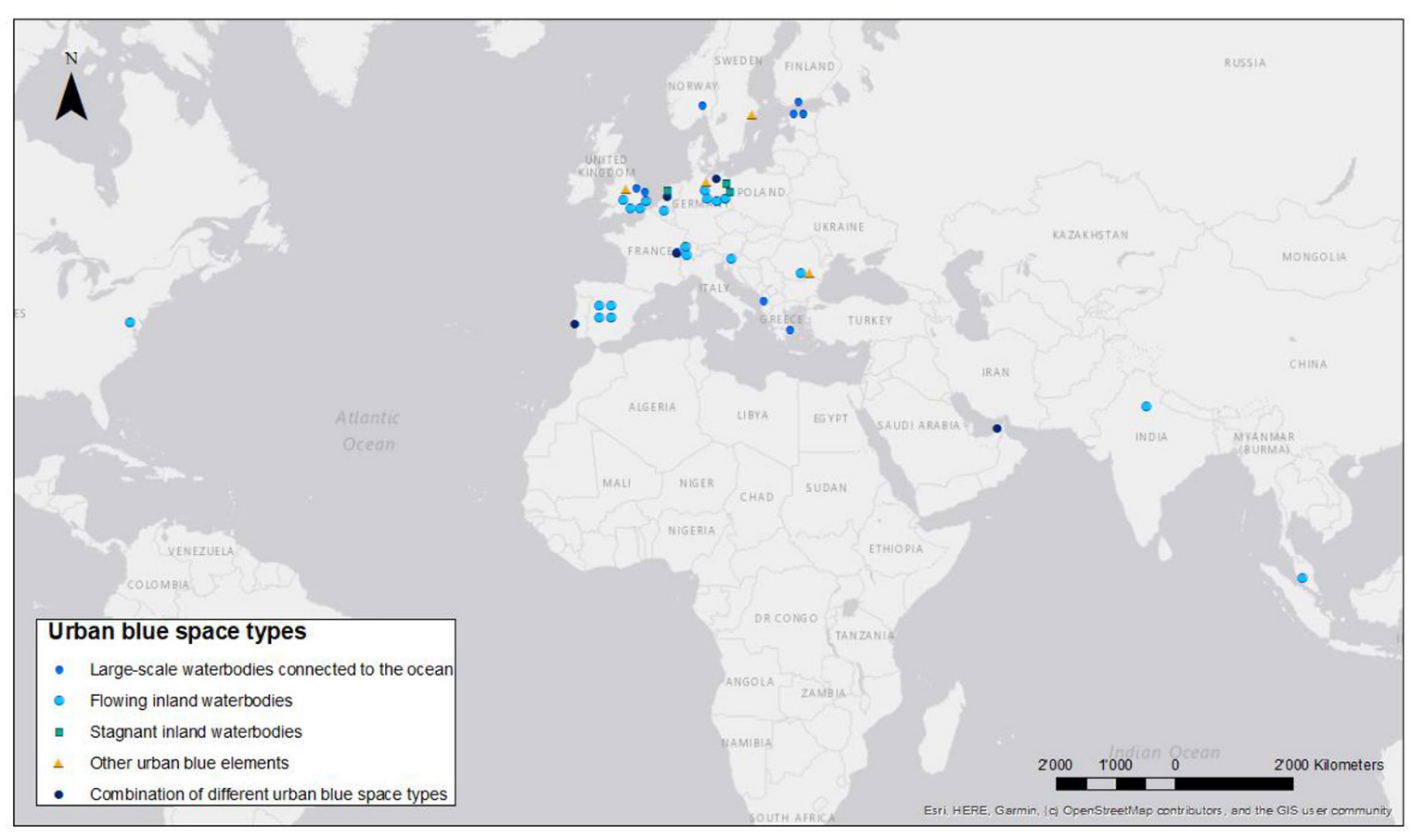
Geographic distribution of the projects under study according to urban blue space types (Note: point symbols refer to the capitols of the project countries and do not reflect the project locations).

The majority of projects (*n* = 35) are located in Europe (see Figure 3). In total, the 39 projects cover 21 countries^2^, with few examples (*n* = 4) from lower- and upper-middle-income countries (LMIC/UMIC). Very small (less than 50,000 inhabitants; *n* = 8), smaller (up to 100,000 inhabitants; *n* = 10) and medium-sized (between 100,000 and 500,000; *n* = 12) cities are mostly represented in this review. Only few examples (*n* = 5) are based in global cities with more than 5 m. inhabitants (see Table 6 in Supplementary Material). To some extent, the small numbers of projects in large metropolitan areas and in other high-income regions (North America, Australia, Asia) might reflect a selection bias: among others, we searched for projects in databases on EU initiatives, and we excluded revitalizations of industrial used water- and riverfronts that are frequently carried out in larger cities.

Flowing inland waterbodies (mostly rivers) make up half of the projects (*n* = 20). Large-scale waterbodies connected to the ocean form the second largest group (*n* = 8); the remaining are stagnant inland waterbodies (e.g., lakes, ponds, pools), other urban blue elements (e.g., basins, swales, rainwater playgrounds), and combinations of different urban blue types. About two thirds of the projects are natural waterbodies, one third are artificial or a combination of natural and artificial blue spaces.

Information on project costs are widely missing or only refer to partial costs. Out of the 21 projects specifying the costs, most involve investments of several million euros. The results substantiate that urban blue regeneration are often costly projects that can struggle to find sufficient funding and/or require uses with high expected returns (17). However, five initiatives involve a total investment of less than half a million euros. These cheaper projects cover temporary and/or smaller-scale regeneration schemes, e.g., creating access to a river pathway.

Whereas in the 20^th^ century a comparatively well-resourced position facilitated urban renewal, recent projects face austerity policies and municipalities are urged to extend their search of funding (18, 19). Consequently, external funding sources such as supranational public, but also private and charity funds, have gained importance in urban regeneration (2, 14). Despite information on funding sources is only available for about two thirds of the projects, this development trend can be observed also in urban blue regeneration: The majority (*n* = 15) are public funded, including supranational funds by the EU and federal, state, and municipal funds. Ten projects are financed by mixed funds from the public and private sector (e.g., housing or water companies) or the public and third-party sector (e.g., NGOs). One project is completely charity funded. The supranational, national and regional funding programs used relate to regional and urban development (e.g., the European Regional Development Fund, the EU-URBAN Program) and – to a lesser extent – to environment and climate action (e.g., the EU-LIFE Fond). Although additional funding sources are crucial for municipalities to engage in urban blue regeneration, its availability differs considerably. Particularly in cities of Western Europe, the experiences made with neighborhood decline and the ecological challenges arisen by industrialization seem to have promoted the implementation of relatively strong funding possibilities that can support urban blue regeneration.

The earliest project has been implemented in 1990 and the latest in 2019. By the time of this publication, the majority of the projects (*n* = 29) has been finalized; 10 projects are implemented on an ongoing basis and/or data are missing whether projects have been officially completed. Among the projects indicating a duration (*n* = 20), the time span ranges between 2 and 20 years, with a predominance of 6 years or more.

Finally, about two thirds of the projects are prize winners in international, national or regional competitions related to architecture and urban design or have been quoted as good practice examples of urban regeneration, e.g., of European riverscapes revitalization (20).

### Stakeholders and Cooperation

Project initiators and further stakeholders involved are classified into public sector (i.e., supranational organizations, national, regional and local authorities), municipal enterprises and quangos (“quasi-non-governmental organizations”), private sector, and voluntary (third party) sector (e.g., NGOs, local communities, citizen associations and research institutes) [adopted from (13)].

**Figure 4:**
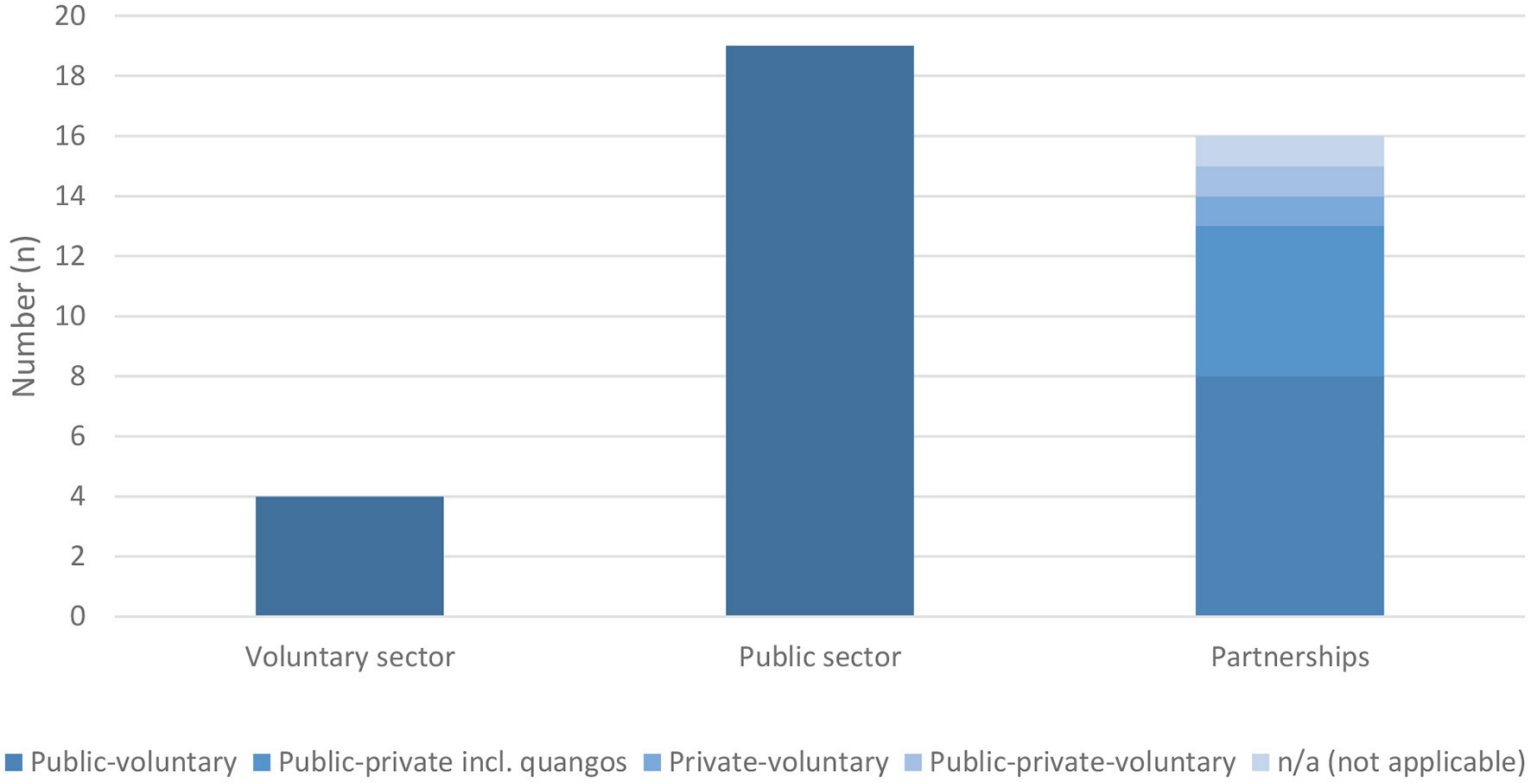
Distribution of project initiators.

As for urban regeneration experiencing a shift from city government to multiple-actor city governance with a rise of partnerships and community engagement (14), most blue space interventions are initiated and implemented by a range of stakeholders. In total 19 projects were initiated by the public sector (mostly by local authorities) and in slightly less cases (*n* = 16), projects were initiated by different kinds of cooperation between the public, private and voluntary sector, including public-voluntary-, public-private-, private-voluntary- and public-private-voluntary-partnerships on the municipal and regional level, with involvement of the central or state government in few cases. Of these two groups, municipal enterprises, quangos or other statutory bodies (e.g., water management associations) sometimes carried out the projects (*n* = 6). Few projects have been initiated by the voluntary sector (*n* = 4), most in the context of political protest, as results of a demonstration or a petition (see Figure 4).

Further stakeholders are involved at different points of the planning and implementation processes and with diverse responsibilities. This applies particularly for the involvement of public authorities, which is largely determined by the local political system and the distribution of power structures. For example, while central, state and regional authorities only function as funders in many projects, their role ranges from administrative supervision and support in planning to holding the ultimate decision power in some other cases. At the municipal level, consultation with different administrative bodies (e.g., the planning, environmental, and economic development departments) is often required. In some projects, cooperation with neighboring municipalities was necessary, as the urban blue regeneration stretched across the administrative borders of the city. In Newcastle-Gateshead [UK-2], this led to setting aside the historical competitiveness between the twin cities and the creation of a new city-partnership. Basically two reasons apply for involving further stakeholders: either because it is mandatory given their jurisdiction or because it is intended that those take over specific tasks and facilitate the implementation. Finally, land ownership is an important factor in decision-making. In L'Hospitalet de Llobregat [ESP-2], for example, after protest of the local community, municipal and regional authorities approved to regenerate the river access, but first needed to sign an agreement with the central government as the landowner. In Ahmedabad [IND-1], the state government owns the riverfront land, thus the state chief minister could exercise power over the project, irrespectively of the acting local government.

As the regeneration of blue spaces can have diverse impacts on the built and social environment, various sectors are involved in the project negotiations, e.g., agriculture, nature protection, regional and urban planning, tourism, transport, and water. However, only few cases (*n* = 7) involved the health sector and the representatives for public health interests and the motivations for involving them vary greatly. In five cases, professional public health leaders [NOR-1] or public health scientists (UK-6; NLD-1; ESP-4; EST-2) were involved in the planning and design phases and in the project evaluation. These examples can be seen as policies that are primarily driven by health rather than by other planning purposes and were thus marked as “public health interventions” (in the following: PHI). In the other two projects, a municipal health department carried out a health impact assessment (HIA) [USA-1] and a health insurance company offered health promotion measures at the regenerated blue space [GER-6]. However, in these cases, health was not the leading planning rationale. In contrast, for example in NOR-1, the intervention was based on evidence collected by the municipality in collaboration with a research center for health promotion and aimed for increasing physical activity, social interaction and participation among the population, preferably marginalized groups.

As a special form of partnership, the exchange of expertise was a common cooperation approach used across the projects reviewed. Expertise exchange relates to skills and knowledge transfer, capacity building, exchange of best practices and scientific contributions (13). Project examples include the formation of technical advisory groups and committees (e.g., UK-1; USA-1) and the consultation of private or third-party experts for advice on environmental, social, and design questions (e.g., UK-3; ARE-1; ESP-2; Fin-1), and/or for conducting feasibility studies and other assessments (e.g., GER-1; ESP-4).

Particularly in large-scale and multi-stakeholder projects, steering committees or independent directive boards were usually responsible to provide the strategic direction. However, in some cases, the responsibilities were less clear, with advisory services delegated to private stakeholders such as architects and design firms (e.g., ALB-1; MYS-1). The enhanced involvement of the private sector in urban regeneration (e.g., as project initiators, funders, or cooperation partner for expertise exchanges) and its effects on cities has been subjected to controversial discussion in recent years: Whereas many writers fear a loss of control by the public sector (14), other scholars take a more balanced position. Accordingly, Short (4) notes that “the neoliberal city is as much an imagining as it is a reality” (p. 532) and argues that the shift to a more entrepreneurial city –as any political movement– has its limits. While the power granted to the private sector has been extensively criticized in research about waterfront revitalization as it often leads to commercial-driven, discriminatory developments that override community needs (7), the results of this study show that the private sector can also support planning visions. For example, projects report that the collaboration with housing companies promoted wider housing improvements, which eventually increased the tenancy rate. Particularly in cities experiencing urban shrinkage, such cooperation could help to prevent further neighborhood decline (17). On the other side, the danger of business-driven developments and related risks of social exclusion and gentrification were identified in a large number of the projects. It therefore seems fair to say that the involvement of the private sector in urban (blue) regeneration is not a black-white binary, but challenges cities to ensure that social/health interests are not undermined by rising economic motivations.

Since raising awareness among the general population and the stakeholders involved has been identified as an essential cooperation approach in controversial projects such as urban green space interventions (13), we investigated how common tools have been applied in urban blue regeneration. As Table 2 shows, all main approaches to building awareness among stakeholders (i.e., environmental education, promotion of sustainable land use and tourism, capacity building, and awareness raising in institutional settings and planning instruments) are also applied in urban blue regeneration.

**Table 2:** Tools to building awareness among project stakeholders [modified from (13)].

**Approaches to build awareness**	**Target groups**	**Examples [project ID]**
Environmental education	Educational institutions, local communities, tourists, wider public	Installation of information boards displaying the local wildlife [NOR-1] Planting actions with local community [UK-3] Environmental education programs, e.g., for kindergartens, schools [GER-6, PRT-1] Awareness raising campaign [ROM-1]
Promotion of sustainable land use and tourism	Fishermen, local (tribal) communities, tourists and tourism operators	Designation and restoration of (tribal) fishing areas [PRT-1, USA-1] Designation of regional park, nature reserves and trails [CHE-3, UK-1, NOR-1] Installation of and improvements to slow mobility and recreational infrastructure, e.g., cycling and walking paths [CHE-1, GER-2, GER-6, ESP-1, ESP-3, UK-4] Installation of nature-based solutions [GER/SWE-1, UK-7, ROM-2]
Capacity building for experts/stakeholders	Practitioners and further stakeholders involved in planning and implementation, e.g., partner organizations	Development of design benchmarks guidance to promote best practice in all affiliated projects [UK-1] Training and information measures, e.g., advisory services [ALB-1, ARE-1, GER-3] Provision of welfare work (e.g., employment and qualification programs) [GER-6, GER-7]
Awareness raising in institutional settings and planning instruments	Regional and local authorities, project partners	Adoption of a riverfront development concept as a binding agreement for all local authorities [CHE-3] Development of (informal) masterplans and integration into official policy [UK-1, GER-6, NLD-2]

### Policy Context

Almost two thirds of the projects (*n* =23) are individual schemes that were not linked to other local approaches or policies implemented elsewhere. The project size ranges from single interventions to larger, integrated umbrella projects that contain few or hundreds of subprojects. One third of the cases form part of policies and programs on the municipal, regional, state, or federal level (e.g., of integrated urban development or regeneration strategies, regional action plans, river- or seafront planning tools), or of research projects. Some are initiated on occasion of certain events, e.g., festivals or architectural exhibitions.

Except the five PHI, however, no project is declared as or linked to a health policy or program. The PHI all form part of EU-funded, multilateral and intervention-based research projects: Four schemes belong to a research program on the links between urban blue spaces, climate and health (15), the other one forms part of an health and environmental research project (INHERIT) that sought to develop and test inter-sectoral innovations for improved public health. In addition, the project was embedded in the municipal development plan and served as a new model for the implementation of evidence-based public health actions.

**Figure 5:**
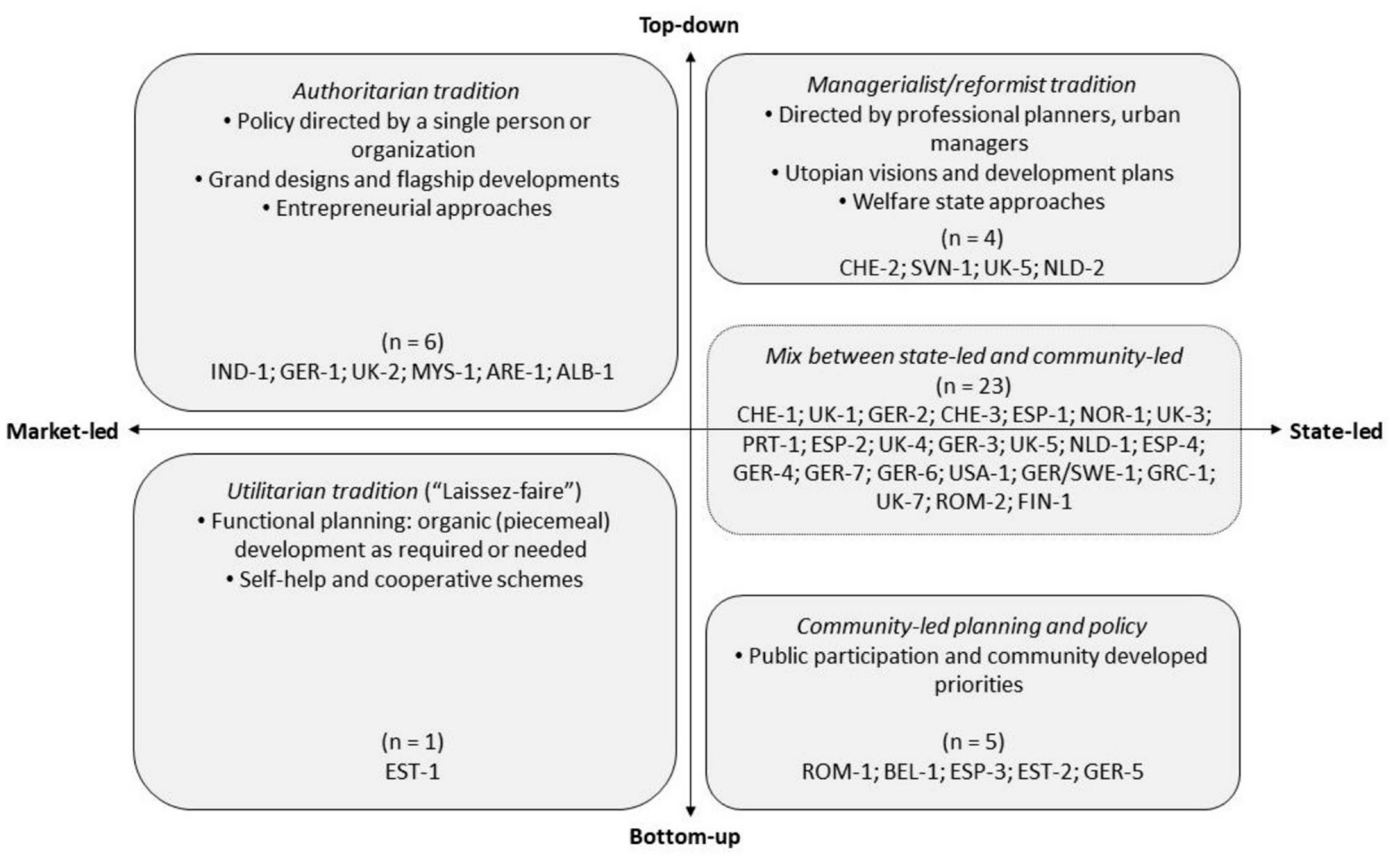
Four broader strategies of urban planning and policy [modified from (14)].

Planning policies can be located on different positions of a multidimensional continuum between market-led and state-led urban development and between bottom-up to top-down implementation [(14); see Figure 5]. For example, urban blue projects might be planned as highly coordinated, consolidated actions (in the authoritarian or managerialist tradition), or might rather be haphazard (e.g., community-led) undertakings.

As a consequence of the changes in urban governance in past years, public participation has become an accepted tenet of practice in urban regeneration (2). However, citizens' involvement in planning and design, and their impact on final decision-making varies greatly: from information and little public impact to empowerment and high public impact (see Table 3).

**Table 3:** Classification of participation in urban development [modified from (21) based on Wouters et al. (22)].

**Category**	**Information**	**Consultation**	**Collaboration**	**Empowerment**
	**Increasing level of public impact**			
	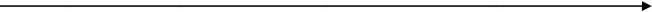			
Description	Citizens inform themselves or are being informed of current plans, decisions, and actions	Citizens are asked to give input and feedback (user-centered design)	Citizens and other stakeholders actively work together in decision-making (co-creation)	The authorities implement the decisions of the citizens
Promise to the public	“We will keep you informed.”	“We will keep you informed, listen to and acknowledge concerns and aspirations and provide feedback on how the public input influenced the decision.”	“We will work with you to formulate solutions together and incorporate your advice and recommendations into the decisions.”	“We implement what you decide.”
Relation	One-way (authorities to citizens)	Limited two-way	Advanced two-way	One-way (citizens to authorities)

Information on participation is available for 29 projects; however of varying depth. In the remaining projects, data is missing or participation has not taken place. As Figure 5 shows, the majority of the projects (*n* = 23) are a mix between top-down and bottom-up schemes. Five projects are community-developed initiatives that exemplify a truly empowering approach: either because those are initiated by communities or because the planning process followed the community's priorities by delegating decisions to them (e.g., vote for design proposal).

Generally, several different ways of how local communities have been engaged can be identified: direct participation (open to all community members), participation through multipliers (e.g., citizen associations, local tenant advisory board), and through cooperation with local institutions, e.g., schools. In many cases, participation has been outsourced, e.g., by commissioning the neighborhood management or third parties. Participation has been carried out at different stages of the planning process, e.g., before or after developing a masterplan.

As seen across the projects, different types of media and tools are used to inform the public and to ensure continuous dialogue, e.g., set up of information points, mailing of information sheets, social media, and information events such as neighborhood walks. The majority of projects use participation instruments ranging between consultation and collaboration, including consultative and collaborative meetings (e.g., round table, neighborhood conference, design and visioning workshops, planning-for-real exercise), surveys, polls and voting tools (e.g., distribution of comment cards, focus groups, interviews, panels), and contests (e.g., citizen idea contest). Some projects might not count as empowerment by definition, but are noteworthy given their highly collaborative character. Those outstanding examples include the civic participation process “Werkstadt Basel” (CHE-1), the co-design process “London's CLEVER action lab” (UK-7), and the community engagement program using “people's panels” (UK-1).

Participatory urban planning can be linked to different aims. In the projects reviewed, these are: to identify community needs and priorities, to set up development goals, to carry out joint activities (e.g., planting actions, construction of furniture), to raise community pride, to facilitate decision-making, and to take care of the needs of certain population groups (e.g., children, youth, people with low socio-economic status). In few cases, participation continued after implementation, e.g., community volunteers who took over management tasks (e.g., GER-5).

Although this review indicates that a mixed (top-down and bottom-up) planning approach with relatively high levels of participation dominates in urban blue regeneration, results should be interpreted with caution: in almost all cases, the data (e.g., number of citizens involved) –if available at all– comes from the project management; thus, it remains widely unknown to what extent communities have been truly engaged. While the rise of partnership models might have strengthened local accountability in urban regeneration, criticism still persist that approaches remain top-down in implementation and that community participation is much restricted by those in power (14). Presumably, the same applies for many urban blue space interventions.

### Project Set-Up and Implementation

All projects are located in or close to deprived residential or mixed-use urban areas, e.g., working class neighborhoods suffering from industrial decline, social housing blocks or derelict city centers. While the social situation prior to the intervention is comparatively similar across the projects (given the pre-defined inclusion criterion of urban area deprivation), the environmental conditions are diverse, including:

- Flood-prone areas, industrially used waterbodies, historical flood defense schemes (e.g., open sewer canals, channeled rivers; partly heavily polluted and deteriorated), neglected beaches and undeveloped coastlines, and a shut-off swimming pool,
- Blue-green public spaces with little amenity (and ecological) value,
- Urban brownfields and wasteland (e.g., abandoned traffic infrastructure).

As such, the access to and the usability of the blue space pre-intervention varies widely, from not having any or only visible access up to utilizable, but outdated public facilities.

Based on classifications of the motivations beyond urban greening interventions (13, 23), the drivers and objectives of urban blue regeneration can be distinguished according to several main themes that evolve from economic, environmental and social urban problems (see Table 4). However, it is important to consider that in practice, the rationales for urban greening – and so for urban blueing – frequently interact and overlap; thus there is hardly only one objective targeted (17, 23).

**Table 4:** Objectives for urban blue regeneration [modified from (13, 23)].

	**Objective**	**Description**
*Environmental*	*Biodiversity conservation*	Actions on combating biodiversity loss by protecting and improving areas of high conservation value, restoring new areas of habitat, and improving connectivity between blue-green spaces (e.g., blue-green corridors)
	*Climate change adaptation and mitigation*	Actions aiming to enhance urban ecosystems' resilience and functioning, e.g., flood management and coastal protection; implementation of NBS for improved urban water management
	*Eco-health and environmental sustainability*	Actions aiming to preserve ecological health, e.g., comprehensive restoration of aquatic ecosystems such as river renaturalization
	*Water quality and supply*	Actions on water purification and regulation such as water treatments (e.g., oxygenation, installation of wetlands), improvements of riparian vegetation, and the provision of water for agriculture and fishing
*Economic*	*Urban competitiveness and entrepreneurship*	Actions focused on using the economic advantages of blue-green spaces for attracting global investment and skilled labor, promoting economic growth and shaping the city image (“urban prestige”)
*Social*	*Health and wellbeing / quality of life (QOL)*	Two approaches: I. *Aesthetics and beautification*: Using water as an aesthetic object to upgrade the physical environment and to provide pleasure in urban settings (e.g., installation of artificial ponds and fountains) *II. Recreation and leisure:* Provision of blue-green spaces for recreational and leisure opportunities, including blue spaces dedicated to specific sports such as swimming pools
	*Social hierarchy and relations*	Creation and maintenance of (private) blue spaces for social positioning, i.e., to express and reinforce social hierarchies, but also as places to promote sociability and cultivate good social relations (e.g., event locations)
	*Social reform and community-building*	Reform-oriented city visions and actions targeting to reconnect and reconcile with nature in order to improve social welfare in the city
	*Symbolism*	Creation, preservation and staging of blue spaces for religious, spiritual and symbolic reasons, e.g., to provide places for contemplation, religious and spiritual practices, to serve as memorials, or to shape a collective identity

**Figure 6:**
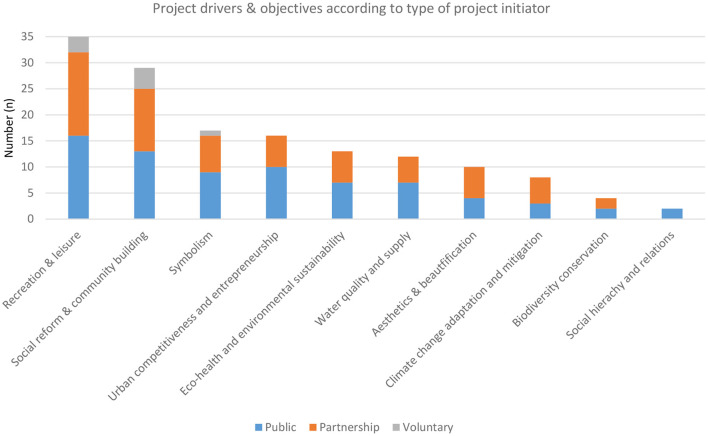
Main themes addressed in the review projects.

The findings confirm that just as recent urban regeneration aims for improvements in several dimensions (2), multiple objectives are linked with the regeneration of blue spaces. As Figure 6 shows, the three most frequently addressed themes within the reviewed projects are, in decreasing order: health and wellbeing/QOL (recreation and leisure), social reform and community building, and symbolism. Most of the projects (*n* = 29) target three or more objectives, in total ranging from schemes with two up to eight objectives. Compared to projects initiated by the public sector or in partnership models, voluntary sector initiatives target only few goals, mainly social reform & community building and recreation & leisure.

In urban regeneration, an increasing focus on environmental sustainability has been observed in recent years and is expected to gain importance in future (2). Despite the results indicating that recreational purposes dominate in urban blue regeneration, environmental goals (“eco-health and environmental sustainability” and “water quality and supply”) still range among the top five objectives. In addition, efforts such as planting actions and environmental clean-ups are reported in projects not explicitly addressing environmental objectives; which might be further caused by the unfavorable environmental conditions in which most urban blue spaces have been left over the past.

Finally, economic aims and interests have been noted to increasingly go along with urban regeneration (14), a tendency that also manifests within urban blue regeneration. However, while “urban entrepreneurship and competitiveness” represent the fourth most targeted objective, an even higher number of projects aims for social transformations, i.e., “social reform and community-building”. This might be due to the context of urban area deprivation applied as a selection criterion in this study. As such, projects are carried out to improve the conditions in deprived communities.

**Figure 7:**
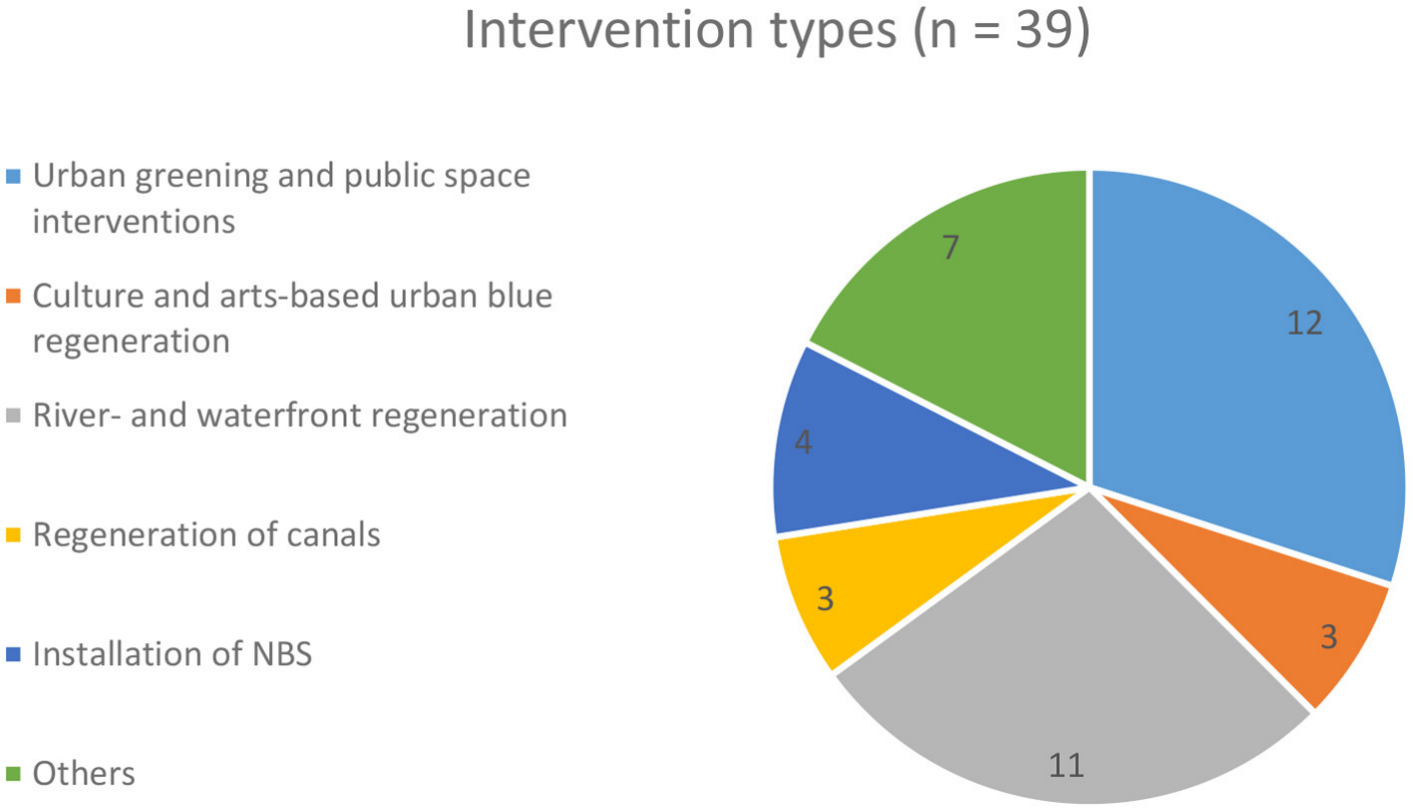
Distribution of projects according to intervention types.

Based on the actions undertaken, the projects can be classified into different types of interventions: a. urban greening and public space interventions (involving blue spaces), b. culture- and arts-based urban blue regeneration, c. river- and waterfront regeneration, d. regeneration of canals, e. implementation of NBS (water-sensitive urban design) and f. others (see Table 6 in the Supplementary Material). As Figure 7 shows, the three most common types are urban greening and public space interventions, river- and waterfront regeneration, and others (e.g., waterside estate action, holistic neighborhood upgrading programs). Special forms of urban greening and public space interventions include the transformation of wasteland and urban infrastructure into blue-green spaces and the redevelopment of urban beaches and coastlines. River- and waterfront regeneration projects can be further distinguished into “classical river-/waterfront regeneration” (large-scale and multipurpose projects) and “community-oriented riverfront regeneration” (limited to a particular neighborhood and focused on improving its accessibility/connectivity) (see Table 6 in the Supplementary Material). Common measures include the provision and enhancement of public use along the blue space (e.g., by installing cycling and pedestrian paths or public features), linking new to old uses (e.g., adding recreational opportunities such as swimming), and waterside (residential, cultural, commercial) developments. In many cases, urban connectivity forms a central theme as the blue spaces –prior to the intervention– represented a major barrier for the surrounding neighborhoods to adjacent communities and the city center, or to local green spaces. Consequently, these projects aim to improve urban connectivity, e.g., by adding pathways, constructing bridges, and changing street alignments.

Environmental changes can be classified into changes to urban blue space quantity (i.e., installation of new blue spaces, transformation of private or undeveloped land into publicly accessible blue spaces), changes to urban blue space quality (i.e., improvements to access, accessibility, and design of existing blue spaces), and changes to the blue space surroundings (e.g., housing improvements, wider neighborhood upgrading). Changes to the blue space quality are most represented (*n* =36); either as single interventions (*n* =12) or linked to changes to blue space quantity (*n* =6), to changes to the surroundings (*n* =10), or to both (*n* =8). The latter case (involving all types of changes) usually applies to larger-scale projects.

While the spatial scale of urban regeneration has increased over the past years and is said to be determined at a city or city-regional level in future (2), the majority of these projects is located on the neighborhood scale (*n* = 21) and in slightly less cases on the city-district scale (*n* = 15). Only few cross the administrative city borders and are found on the metropolitan regional scale (*n* = 3).

Almost all interventions are permanent; only two projects make temporary changes to the environment. In three projects implementing NBS, environmental changes are permanent; however, water provision is impermanent, dependent on local precipitation patterns.

### Outcomes and Impacts

In total 20 projects provide information on evaluation. Ongoing or pre- and post-intervention evaluation is the most common case (*n* = 11), followed by evaluation only before (*n* = 5) and only after project implementation (*n* = 4). In the case of pre- and ongoing evaluation, assessments have been carried out to identify attraction points, users' characteristics and needs [e.g., ESP-2, NOR-1], and to refine the planning by highlighting intervention priorities [e.g., UK-1; GER-6].

The type and level of evaluation varies greatly across the projects. If specified, types include rather descriptive case reports, cost-benefit analysis, technical and design studies, needs and impact assessments (e.g., environmental, health, social), and project-specific evaluation tools and quality assurance schemes. Subjects of the evaluation range from the measurement of single actions and subprojects (e.g., performance of the neighborhood management implemented), of economic, social and environmental performance indicators (e.g., water quality), to changes in blue space user behavior and health effects (e.g., physical activity). In most cases, the evaluation was carried out by project partners, e.g., research institutes, or by project initiators.

As Table 5 shows, achievements and benefits of urban blue space regeneration can be classified into physical improvements, ecological, economic, social, and other benefits. Most benefits were documented after project implementation; however, few projects provided only anticipated outcomes since the implementation is not yet completed.

**Table 5:** Achievements and benefits in the projects reviewed.

**Type of outcome**	**Achievements and benefits reported**
Physical improvements	Creation of public space and improved access to urban blue and green
	Enhanced quality of urban blue spaces, e.g., cultural and recreational facilities
	Aesthetical upgrading, e.g., by high-quality architecture and wider neighborhood
	Transformation of spatial structures and improved urban connectivity
	Creation of blue-green corridors and open space nets (improved spatial integration)
Ecological benefits	Improved water and ecological quality, e.g., enhanced biodiversity
	Improved stormwater management and flood prevention
	Renaturalization of aquatic ecosystems and restoration of brownfields and wasteland
	Reduction of environmental pollution, e.g., emissions
Economic benefits	Improved regional, city or neighborhood image
	Increased numbers of visitors
	Increased land and property values in the regeneration area
	Employment and training creation
	New location advantages (venue marketing)
	Attraction of investment and commercial growth in close by areas
	Increased municipal income
	Reduction of operating costs and rents through improved stormwater management
Social benefits	Improved local identity and belongingness to place (“sense of place”)
	Revived urban areas; improved quality of urban life
	Re-balancing the social structure of the local population
	Promotion of social interactions, increased community engagement
	Improved social cohesion and improved inclusion of vulnerable populations
	Reduction of user conflicts
	“Democratizing effects”: Stimulation of urban (blue) development debates (e.g., on the risk of gentrification)
	Support of environmental education, e.g., provision of outdoor classrooms
Effects on public health	Behavioral changes toward healthier lifestyles, e.g., increased physical activity
	Healthier urban environments, e.g., reduced air and noise pollution
	Health policy changes, e.g., integrated action on public health
Other benefits	Interventions as drivers for general site improvements, further policy action, and enhanced local cooperation
	Interventions as models for the implementation of evidence-based public health actions or to test urban regeneration approaches
	Dissemination of knowledge in urban (blue) regeneration and green technologies

Beneficial changes to the physical environment include more than just improving the access to urban blue spaces and/or creating high-quality blue spaces. As such, larger projects (e.g., CHE-1, PRT-1) usually involve wider neighborhood investments, improvements to urban connectivity and the spatial integration of blue-green infrastructure.

Ecological benefits relate to improved (storm-) water management, flood control, enhanced water and environmental quality, increased biodiversity and local animal populations (e.g., CHE-3), and wider ecological recovery (e.g., re-naturalization of entire river systems) (e.g., GER-3, GER-6). By improving urban connectivity and increasing the number of active transport users, some projects contributed to the reduction of environmental pollution such as emissions from urban traffic (e.g., ESP-1, NOR-1).

Given their qualities as highly preferred tourist destinations and places of residence, urban blue spaces are known to entail considerable economic advantages. Following this, increased land and property values belong to the frequently mentioned – and according to results of a cost-benefit analysis (NOR-1) – to the most cost-effective outcomes. Economic benefits further include an improved local image, increased numbers of visitors, employment creation, new location advantages, commercial growth in the surroundings, increased municipal income, and reduced operating costs and rents through improved stormwater management.

Finally, a range of social benefits is reported across the projects. In many cases, the regeneration of “the blue” not only functions as a physical but also a symbolic reconnection to urban waters, which provide emotional benefits for people. Among others, “the regenerated blue” creates a new or improves the local identity and belongingness to place [“sense of place”, (24)]. As a strategy against urban sprawl, urban blue regeneration contributes to revive city centers or rundown neighborhoods; thus improving the urban quality of life. In many cases, this is followed by the attraction of higher-income people and consequently, a re-balanced social structure of the local population. Further social benefits include the reduction of user conflicts and enhanced social interactions between different population groups, improved social cohesion and inclusion of vulnerable populations, and increased community engagement. Particularly in highly participatory or bottom-up projects, urban blue regeneration enabled people to voice their concerns and highlight deficiencies of current urban planning (“democratizing effects”) (25).

Other benefits relate e.g., to the dissemination of knowledge on urban (blue) regeneration and green technologies, to enhanced local cooperation, and to subsequent policy action such as the implementation of neighborhood management on the city-level (CHE-1).

The projects indicate multi-dimensional effects on public health that can be summarized as i. behavioral changes toward healthier lifestyles, ii. healthier urban environments, and iii. health policy changes.

First, health outcomes arise from the increased use of blue spaces as leisure, activity, and social spaces. As urban blue spaces are more accessible and/or more attractive, many projects (*n* = 27) have noted increased user numbers and behavioral changes of the local population, e.g., increased physical activity levels and social interactions, which is known to impact people's physical and mental wellbeing. While most of the projects report that users are more engaged in moderate-to-vigorous physical activity, it is also likely that the improved amenity value of blue spaces increases sedentary activities. In Montcada i Reixac [ESP-4], for example, a significant increase of visitors engaged in sedentary, moderate, and vigorous activity in the renovated area was noted. The evaluators conclude that the increase of physical activity could prevent premature morbidity and mortality significantly and that the intervention could save the healthcare system several million euros (26). As noted in previous research [e.g., (5, 27)], social benefits such as improved social cohesion might belong to the most important health effects of blue spaces. Projects in this review confirm that social interactions not only increased, but that blue space users also diversified post-intervention, e.g., including children, elderly people, women, and ethnic minorities.

While it is obvious that urban blue regeneration can directly improve the health and wellbeing of blue space users, non-users might benefit from such interventions indirectly, e.g., by improved environmental quality or by the economic advantages blue spaces entail.

Lastly, the projects indicate that urban blue regeneration can lead to beneficial changes in urban planning and policy, e.g., an increased awareness and consideration of environmental justice, the development of subsequent policies, and integrated action on public health such as the provision of health promotion measures (e.g., walking and cycling tours).

Overall, beneficiaries in the reviewed projects – both, intended and documented – represent a range of actors, including local communities, tourists, businesses, communities of interest (e.g., cyclists, walkers), local authorities, and urban regeneration specialists.

In contrast to the range of positive effects reported across the projects, comparatively little is said about negative outcomes. However, drawbacks and trade-offs involve diverse environmental and social problems that could significantly reduce the health potential of blue spaces. From an environmental perspective, by becoming a new “urban hotspot”, the rise of visitors resulted in increasing urban stressors such as air and noise pollution (GER-2) or is expected to potentially lessen environmental benefits gained in the long run (NOR-1). In implementing NBS for improved stormwater management (GER/SWE-1), the lack of experience with such systems led to construction failures and inappropriate design (e.g., oversized gutters). In consequence, littering and a lack of maintenance devalued the blue spaces. In Cologne and Ahmedabad (GER-1; IND-1), criticism involves the lacking consideration of environmental impacts in favor of urban development. As such, unsustainable practices are promoted that cause and aggravate environmental hazards (e.g., flooding, water scarcity). Short-term thinking has further been reported in terms of insufficient funding and maintenance post-intervention (e.g., UK-1).

Social drawbacks and trade-offs include the intensification of user conflicts and the creation of hotspots for anti-social behavior (e.g., CHE-1, CHE-3). In some cases, it is even questioned if the project was truly worth the investment as social problems continue to exist or even worsened (e.g., due to gentrification) or led to a steep increase of the city's public debt (e.g., ESP-1; UK-2). Most common problems however, relate to questions of environmental justice: some projects are criticized to endanger social inclusion, to discriminate and displace vulnerable groups and to re-inforce existing socio-spatial inequalities (e.g., IND-1; USA-1). Following the increase of land and property values, gentrification is considered as a particular challenge in the long run (e.g., GER-2; FIN-1) and critical questions are raised on how to prevent it effectively.

Finally, projects report drawbacks during planning and implementation, e.g., a temporary worsening of urban traffic, extensive assessments, bureaucratic and time-consuming processes of coordination, limited participation, conflicts with stakeholders and political opposition. While the latter often leads to delays and impose further conditions to the project, it can actually turn positive, e.g., if further environmental assessments are carried out due to the protest of environmental charities (ESP-1), or if areas are left undeveloped for nature protection (FIN-1). On the downside, it can leave projects in abeyance, e.g., if construction is stopped after a change of government (ESP-2).

Across the projects, several factors are reported that facilitated the planning and implementation process. To mitigate adverse impacts and improve the strategic planning, applying impact assessment tools such as HIA or SIA pre-intervention is considered useful as it provides important information on present water and land uses, on the health-related and social aspects of blue spaces, and on potential effects of the regeneration that can be integrated into policies and design plans (28). As such, applying a HIA can help to promote a “more comprehensive protection of public health” [(29) p. 318]. For example, by following the recommendations of the HIA, planners in Minneapolis (USA-1) were encouraged to incorporate opportunities for recreation and public access, which could contribute to reducing health disparities.

Although it demands a sincere willingness to cooperate and makes organization more complex, cross-sectoral partnerships are seen as a main success factor that can allow implementing a range of subprojects within a short timeframe. Particularly in projects with many stakeholders, coordination can be facilitated by setting up a steering committee (e.g., UK-1) that works as the hub of the network.

The active participation of citizens not only facilitated the implementation and ensured community-oriented blue space design, but also fostered neighborhood relations in many projects. As such, a high level of participation and bottom-up approaches contribute to successful urban blue regeneration; however, it requires political will and joint efforts to implement community-led initiatives. As participation relies much on the support from public bodies, projects in this review highlight the added value of working with local neighborhood management and third parties. Yet, difficulties still arise, e.g., to involve the private sector such as housing companies or socially vulnerable populations.

Land ownership, legislations, funding, and challenges related to cross-sectoral cooperation are major barriers that arise across the projects. The experiences indicate that a high proportion of municipal land facilitates an early construction start. Conversely, complex ownership relations can hinder urban blue regeneration and make such projects unrealizable. Despite possibilities are specified that facilitate funding (e.g., pooling of funds, applying for urban development programs), the economic situation of cities remains a barrier for urban blue regeneration and economic recession can compromise the implementation at any time.

As noted earlier, difficult coordination requirements often complicate the planning and implementation process. Given that urban blue regeneration usually involves a range of stakeholders in various sectors, contradictory relations and the necessity to negotiate interests is rather the norm than the exception. Common conflicts evolve around competing claims of public vs. private uses and the integration of public health, social-economic (e.g., recreation, urban expansion) and ecological interests (e.g., flood control, nature protection). Nevertheless, many projects show that it is possible to reconcile public and private uses as well as ecological and recreational interests; for example, by zoning rivers and lakes into different areas for public swimming and wildlife habitat. Indeed, the integration can even facilitate the maintenance of blue spaces, e.g., as reported for transforming a swimming pool into a natural bath (GER-5). Yet, the demands put on landscape design are complex. In the case of stormwater management systems, water hardly functions as a single design element and solutions that account for the small size and temporal availability of water need to be found. To facilitate such interventions, the creation of a profound knowledge base is considered necessary (8).

### The Role of Public Health in Urban Blue Regeneration

Despite the major role blue spaces could play for public health promotion (30), the results of this review indicate that public health hardly plays an explicit role in urban blue regeneration. As such, public health professionals are barely involved in the projects or assigned only with selected responsibilities, e.g., conducting a HIA. The PHI identified have in common that they are mostly smaller-scale projects (“urban greening and public space interventions”) on the neighborhood level, planned in public-voluntary-partnerships in the context of larger research programs, with high community participation. As a distinctive feature, most have done detailed (pre- and post-intervention) assessments, e.g., on user characteristics and behavior, community needs, and health effects. Compared to other projects, it is noteworthy that those hardly report any drawbacks after implementation, especially no negative effects regarding social dimensions.

Although public health is implicitly targeted in many other projects (e.g., aiming for improved recreation and leisure opportunities), actors such as health departments are not involved and it remains unclear which priority is given to health among other objectives. For example, while improving public health through implementing NBS is a key aim in two projects (UK-7; ROM-2), it remains unclear how this aspiration ranks among other goals, e.g., to increase economic opportunities. Finally, this points to a commonly known problem: health as a universal policy objective has been increasingly pursued in inter-sectoral approaches [“health in all policies” (HiAP)] to address the powerful social determinants of health. Yet, among the various values placed on urban planning, health as a political priority still needs to be negotiated (31, 32). Accordingly, reconciling the competing demands of diverse stakeholders in urban blue regeneration continues to pose a planning challenge, in which health is rarely the leading rationale and guiding design principle (33).

Conversely, the results of this review show that urban blue regeneration can still positively affect public health even if not being conceptualized as a PHI. However, and particularly in larger-scale projects, the danger of adverse impacts on health and wellbeing of local communities or population groups is too high to leave urban blue regeneration to chance. To maximize the health-related benefits of urban blue regeneration and to prevent socially exclusive developments that might arise from increasing business-driven approaches, project managers should consider involving the health sector more actively in planning and implementation. For example, as the public health service is usually not a statutory consultee in planning, information of local health departments about urban blue regeneration proposals would give them the opportunity – based on the resources available – to assess health-related impacts and recommend potential mitigation strategies. Public health professionals are further experts in local health promotion and prevention needs. Therefore, they can point to intervention areas and link projects to health behavior measures. Overall – and as shown in the PHI in this study – collaborating with public health experts from science and practice can facilitate evidence-based, integrated and highly accepted urban blue regeneration and ways to achieve such. From the authors' perspective, this is especially important since public health professionals are trained to apply a population focus based on interdisciplinary considerations (34) and can therefore account for the complex ways in which environmental interventions affect human health. As such, they are also suited to consult with experts such as environmental scientists to achieve integrated (“One Health”) effects, i.e., urban blue spaces that benefit animal, human, and environmental health likewise. As seen across the reviewed projects, urban blue regeneration entails considerable ecological benefits, although these are only visible in the long run. Yet, research has shown positive correlations between the environmental quality of blue spaces (e.g., biodiversity, level of pollution) and the health benefits people derive from blue space visits (10). Without the involvement of public health experts, it should at least be questioned in each urban blue regeneration project whether health aspects are adequately reflected and who can be held responsible for advocating and assessing these.

### Recommendations for Practice

Regenerating blue spaces can bring about multiple benefits to cities that directly or indirectly promote public health. As such, most of the projects in this review report that despite all barriers, urban blue regeneration is economically feasible and profitable from a societal and environmental perspective, particularly in deprived communities that usually lack such resources. This also hold true for smaller and even impermanent blue spaces, for which projects in this study demonstrate comparable (economic, ecological and social) outcomes. Stormwater management –if being well designed– can significantly enhance the experience of urban spaces and can be a way of bringing “the blue” into communities without access to waterbodies. Despite clear variations in the project outcomes and the benefits obtained among different stakeholders, we support that urban blue regeneration should be more explicitly promoted as an environmental health intervention. As noted earlier, public health representatives could be able to make valuable contributions to such schemes, to exert influence on critical aspects and to take over specific tasks, especially those planners are usually not trained in. Finally, a stronger recognition within public health might enhance funding possibilities, e.g., if using means provided for disease prevention and health promotion. For example, in Germany, health insurances are obliged to annually spend a certain amount per assured person for health promotion and prevention. As seen in some projects, temporary installations are a cost-effective strategy to achieve short-term improvements and to test urban blue regeneration, which could then be converted into permanent structures.

This review has shown that many challenges are associated with urban blue regeneration. Generally, the barriers identified are consistent with findings from urban waterfront developments [e.g., (17)] and the implementation of NBS [e.g., (35)] and include the following aspects: funding, complex coordination requirements, land ownership, public acceptance, competing claims and land-use conflicts. Certainly, the challenges posed to cities vary from place to place and the opportunities to overcome those differ, as well as “(…) different priorities will be agreed and implemented” [(2) p. 329]. However, several factors have been identified that facilitate urban blue regeneration.

As several projects report that future funding and maintenance was not adequately considered in advance, a realistic assessment of funding and maintenance needs seems critical to protect the benefits gained. Flexible financing schemes (e.g., having several funding sources, uncommitted subsidy funds) have shown to be crucial. Despite the global competition of cities for attracting foreign investments (18), projects in this review prove that supranational funds can be still a promising funding source to regenerate blue spaces, e.g., when linking such schemes to transnational research programs or to integrated action on environmental sustainability and climate protection. To further overcome economic constraints, natural design solutions that demand less care, donations, and the workforce offered by volunteers or acquired through employment programs can contribute to reduce costs. Projects have shown that involving local communities and third parties such as sports associations (e.g., GER-5) can be effective ways to set up sustainable maintenance arrangements after implementation; however, those need to be supported technically.

The range of stakeholders to be involved as well as their different demands lead to complex decision-making and planning processes. Yet, evaluators and project managers recommend cross-sectoral partnerships (including community engagement) as they provide the chance to increase public acceptance, to generate synergies for comprehensive and integrated action, and to share responsibilities. From the perspective of municipalities, achieving a broad consent can also reduce the electoral risk when deciding on urban blue regeneration. In the projects reviewed, the involvement of third parties has been noted to enable early knowledge exchange in planning and design, to advocate for community needs, and to maximize health-related benefits. Collaborating with stakeholders from science can support monitoring and evaluation, especially as municipal resources are usually limited. Generally, from the authors' perspective, pre-assessments are not sufficient and longitudinal studies should accompany urban blue regeneration as those could provide data on the long-term impacts and public health effects. This is particularly important since factors such as user behavior or the maintenance of spaces can only be assessed in the long run, as places change over time and are continuously shaped by its users. Financing such research represents a challenge, but the authors would argue in support of transdisciplinary projects involving partners from science who can apply for research funds (like in the projects identified as PHI).

As shown in this review, there is a range of cooperation approaches to building awareness among the stakeholders involved. However, it must be considered that different stakeholders are driven by different motivations and that particularly commercial interests need to be reconciled carefully: unless public use is required by regulation, the regeneration might run into risk to transform blue spaces into “exclusive edges” (7) that endanger social inclusion. Consequently, while urban blue regeneration can help to reduce environmental health inequalities, the economization of urban waters can be a catalyst for “blue gentrifications” (33) and urban inequalities that might compromise or worsen the health of original inhabitants (29). In this review, the risk for gentrification and its effective prevention is discussed controversial: on the one side, projects recommend to ensure public control over the real estate market and to provide mixed housing; however, investment into high-quality architecture is appreciated to promote urban regeneration. Further, local residents might not accept the implementation of social housing, even when they themselves are at risk of being displaced. To some extent, the cultural context and the experience with social housing in urban planning seem to matter. As shown in some cases (BEL-1; EST-1), arts and culture can be effective tools for consciousness-raising and promoting public debates about urban blue regeneration and the risk of gentrification.

In evaluating urban regeneration, partnership, strategy, and sustainable development have been identified as a “troika of approaches that determine and drive successful regeneration” [(2) p. 320]. Accordingly, a comprehensive planning and design approach that aims for multi-functional and sustainable blue spaces is imperative in urban blue regeneration. Evidence from healthy blue space research [e.g., (10, 36)] further highlights the need for blue spaces that provide a high amenity and ecological value, given their interdependencies.

Contrary to the trend of developing homogeneous waterfronts, researchers have highlighted the need for locally responsive design solutions that account for the singularity of each blue space (7). To allow for a systematic consideration of ecological, economic, and social aspects as well as the health dimensions of proposed projects, impact assessments of different kinds (e.g., environmental, social, health) and differentiating between different social groups should be integrated in the planning process. This can be combined with participation processes, e.g., by investigating users' needs concurrently. However, as impact assessments are mostly voluntary processes that demand additional work, the added-value of such tools has hardly been recognized in current water-related planning and policy. Scholars, therefore, point to the importance of awareness-raising and training of decision-makers [e.g., (28)].

Finally, different understandings of deprivation could have led to the selection of intervention areas that not benefited those with the most urgent needs for environmental changes. While a multi-dimensional measurement of regional deprivation has become common in science, concepts vary between countries, making deprivation “(…) relative to what is customary to the societies in which people live” [(37) p. 21]. In addition, urban problems and the areas in which they manifest are not self-appointed, but defined in various ways and the “articulation of causation” justifies policy action (14): “Put simply, urban regeneration is designed to address whatever policy makers or practitioners think, or want to believe, is causing the problems they observe” [(14) p. 144]. Consequently, the intervention areas for urban blue regeneration should be prioritized according to which communities are most vulnerable and projects should be linked to further policies aiming to improve the living conditions in deprived areas.

### Research Limitations

Following the selection process, the projects might be biased toward highly community-oriented and successful (prize-winning) regeneration schemes located mainly in Europe that are not representative for urban blue space interventions in other parts of this world. Many projects are based in smaller cities where e.g., land contestations are usually less pronounced as compared to larger cities. As such, urban blue regeneration here might be less affected by land use conflicts as elsewhere. The restriction to the search terms “health” and “wellbeing” instead of using a wider range of aspects related to health such as “physical activity” could have led to relevant projects being overlooked. The information about the projects usually come from stakeholders involved and could not be validated from external parties. In some cases, the data available was quite limited. As there is mostly cross-sectional data available, project outcomes might have been over- or underestimated. Although we tried to classify projects according to definite criteria and consulted in case of ambiguities, classification bias might have occurred.

## Conclusions

The regeneration of blue spaces is widespread in urban development and planning and brings about multiple benefits to cities, including threefold effects for public health: i. behavioral changes toward healthier lifestyles, ii. healthier urban environments, and iii. health policy changes. In contrast, public health is so far mostly considered only indirectly in urban blue space regeneration. The results indicate that the potential to use urban blue regeneration as a community-based health intervention has yet to be realized. Finally, such projects can improve the access and accessibility to, and the quality of health-enabling places within cities, but entail the risk to reinforce existing urban (health) inequalities. Expanding the so far marginal involvement of public health experts could be one approach to tackle this issue and to ensure that health interests and needs of different social groups are reflected in planning. Based on the projects' experiences, achieving multi-functional blue spaces for different user groups and types of activity, as well as meeting economic, ecological, and social interests is crucial. Although the projects in this study are distinct –in terms of their geographical, cultural, and political contexts, the local capabilities, as well as the interventions undertaken– they provide valuable lessons to be learned and sources of inspiration for future urban blue regeneration.

## Author Contributions

AB was the main author of this paper. TF and TK checked the analysis and reviewed the overall paper, while CH mainly contributed to the paper by developing figures. All authors contributed to the article and approved the submitted version.

## Funding

This research has been funded by the Ministry for Culture and Sciences of North Rhine-Westphalia.

## Conflict of Interest

The authors declare that the research was conducted in the absence of any commercial or financial relationships that could be construed as a potential conflict of interest.

## Publisher's Note

All claims expressed in this article are solely those of the authors and do not necessarily represent those of their affiliated organizations, or those of the publisher, the editors and the reviewers. Any product that may be evaluated in this article, or claim that may be made by its manufacturer, is not guaranteed or endorsed by the publisher.

## Footnotes

^1^Despite different terminologies, urban regeneration usually involves deliberate interventions that aim “[to] bring about a lasting improvement in the economic, physical, social and environmental condition of an area that has been subject to change or offers opportunities for improvement” (Roberts et al., (2): 315). Target areas are often those where social disadvantages concentrate, e.g., communities that experienced serious economic decline (Hall & Barrett, (14); Roberts et al., (2)).

^2^Counting the self-governing commonwealth of Puerto Rico as part of the United States of America.
