# Supplementary material for: The Regeneration of Urban Blue Spaces: A Public Health Intervention? Reviewing the Evidence
Source: Front Public Health. 2022 Jan 13;9:782101. doi: 10.3389/fpubh.2021.782101 (PMC8792750; doi:10.3389/fpubh.2021.782101)
Supplement: Supplementary file 1 [file Table_1.pdf]

## Table 6: General characteristics of projects included in the review

| Project-ID | Project name | City | City size[i] | Year of implementation | Project costs | Funding | Urban blue space type[ii] | Specials[iii] | Main reference |
|---|---|---|---|---|---|---|---|---|---|
| **I. Urban greening and public space interventions (involving blue spaces)** | | | | | | | | | |
| CHE-2 | Leisure center Dreirosen | Basel, CHE | M | 2006 | 11.5 m. CHF | Mixed | Combination of different urban blue space types (A) | ✪ | Montag Stiftung & Regionale2010, 2008 |
| CHE-3 | Swimming spot Oberer Letten | Zurich, CHE | L | 2009 | 1.5 m. CHF | Public | Flowing inland waterbody (N) | ✪ | Montag Stiftung & Regionale2010, 2008 |
| GER-5 | Citizens' park Heilborn | Merzig, GER | XS | 2008 | n/a | Mixed | Stagnant inland waterbody (A) | n/a | BMI, n.y. |
| NLD-1 **(*PHI*)** | Development of a recreational waterfront | Amster-dam, NLD | XL | 2018 | n/a | n/a | Stagnant inland waterbody (A) | n/a | BlueHealth, n.y. |
| ARE-1 | Utilizing Waterfronts as Inclusive Public Spaces | Dubai, ARE | XXL | 2008 | n/a | n/a | Combination of different urban blue space types (A, N) | n/a | Project Public Spaces (PPS), 2008 |
| ESP-2 | The Braided Valley | Elche, ESP | M | 2013 | 2.38 m. EUR | Public | Flowing inland waterbody (N) | ✪ | Centre of Contemporary Culture of Barcelona (CCCB), 2019 |
| *Transformation of wasteland and urban infrastructure (into blue-green spaces)* | | | | | | | | | |
| ESP-1 | Madrid Rio | Madrid, ESP | XXL | 2015 | 4.1 bn. EUR | Mixed | Flowing inland waterbody (N) | ✪ | INHERIT, n.y. |
| NOR-1 **(*PHI*)** | The Malvik Path | Malvik, NOR | XS | 2016 | n/a | Mixed | Large-scale waterbody connected to the ocean (N) | ✪ | INHERIT, n.y. |
| GER-3 | Stassfurt: Relinquishing the old center | Stassfurt, GER | XS | 2010 | 2.9 m. EUR | Public | Stagnant inland waterbody (A) | ✪ | CCCB, 2018 |
| *Redevelopment of urban beaches and coastlines* | | | | | | | | | |
| ALB-1 | Himara Waterfront | Himara, ALB | XS | 2017 | 1.8 m. EUR | Public | Large-scale waterbody connected to the ocean (N) | ✪ | CCCB, 2020 |

| | | | | | | | | | |
|---|---|---|---|---|---|---|---|---|---|
| EST-2 **(*PHI*)** | Enhancing an urban coastline | Tallinn, EST | L | 2018 | n/a | n/a | Large-scale watebody connected to the ocean (N) | n/a | BlueHealth, n.y. |
| UK-6 **(*PHI*)** | Urban beach regeneration | Plymouth, UK | M | 2018 | n/a | n/a | Large-scale waterbody connected to the ocean (N) | n/a | BlueHealth, n.y. |
| **II. Culture- and arts-based urban blue regeneration** | | | | | | | | | |
| UK-2 | From Coal to Culture | Newcastle-Gateshead, UK | L | Ongoing since 1990 | Several m. GBP. | Mixed | Flowing inland waterbody (N) | ✪ | Montag Stiftung & Regionale2010, 2008 |
| BEL-1 | Temporary Bridge over the Charleroi Canal | Brussels, BEL | XXL | 2014 | 50,000 EUR | Charity | Flowing inland waterbody (A) | ✪ | CCCB, 2018 |
| EST-1 | "LIFT11" Urban Installations Festival | Tallinn, EST | L | 2011 | 180,000 EUR | n/a | Large-scale watebody connected to the ocean (N) | ✪ | CCCB, 2018 |
| **III. River- and waterfront regeneration** | | | | | | | | | |
| CHE-1 | Impulse project Rhine | Basel, CHE | M | Ongoing since 1999 | n/a | Mixed | Flowing inland waterbodies (N) | ✪ | Montag Stiftung & Regionale2010, 2008 |
| UK-1 | Mersey Waterfront Regional Park | Liverpool, UK | L | 2020 | 81 m. GBP. | Mixed | Large-scale waterbodies connected to the ocean (N) | ✪ | Montag Stiftung & Regionale2010, 2008 |
| MYS-1 | Kuching Waterfront Re-development | Kuching, MYS | XL | Ongoing since 1993 | 89.9 m. MYR | Public | Flowing inland waterbody (N) | ✪ | Breen & Rigby, 1996 |
| USA-1 | HIA of urban waterway decisions | Minneapolis, Seattle, Rochester, San Juan, USA | n/a | Ongoing since 1990 | n/a | n/a | Flowing inland waterbodies (N) | n/a | Korfmacher et al., 2015 |
| IND-1 | Sabarmati Riverfront | Ahmedabad, IND | GL | Ongoing since 1997 | 250 m. USD | Public | Flowing inland waterbody (N) | ✪ | SRFDCL & AMC, n.y. |
| UK-3 | The London Rivers Action Plan (LRAP) | London, UK | GL | 2015 | n/a | Public | Flowing inland waterbodies (N) | ✪ | The River Restoration Center, 2009 |
| SVN-1 | Rearrangement of Ljubljanica | Ljubljana, SVN | L | 2011 | 20.1 m. EUR | Public | Flowing inland waterbody (N) | ✪ | CCCB, 2018 |

| | | | | | | | | | |
|---|---|---|---|---|---|---|---|---|---|
| | riverbanks | | | | | | | | |
| GER-6 | Together for the new Emscher valley | Ruhr Metropolis, GER | GL | Ongoing since 1992 | 5.38 bn. EUR | Public | Flowing inland waterbodies (N) | ✪ | Emscher-genossenschaft, n.y. |
| *Community-oriented riverfront regeneration* | | | | | | | | | |
| UK-5 | Riverside Masterplan | Castleford, UK | XS | 2008 | 3.65 m. EUR | Public | Flowing inland waterbody (N) | ✪ | CCCB, 2018 |
| ESP-3 | Access pathway to Llobregat River from Bellvitge | L'Hospitalet de Llobregat, ESP | L | 2011 | 204,342 EUR | Public | Flowing inland waterbody (N) | ✪ | CCCB, 2018 |
| ESP-4 **(*PHI*)** | Enabling access to an urban river | Montcada i Reixac, ESP | XS | 2016 | n/a | n/a | Flowing inland waterbody (N) | n/a | BlueHealth, n.y. |
| **IV. Regeneration of canals** | | | | | | | | | |
| ROM-1 | Recovering of the Moirii Canal | Reghin, ROM | XS | 2012 | 390,000 EUR | Public | Flowing inland waterbody (A) | ✪ | CCCB, 2018 |
| UK-4 | Bow Riverside | London, UK | GL | 2011 | 2.9 m. EUR | n/a | Flowing inland waterbody (A) | ✪ | CCCB, 2018 |
| GER-2 | Neighborhood development Jungbusch | Mannheim, GER | L | 2007 | 3.8 m. EUR (partial) | Mixed | Flowing inland waterbody (A) | ✪ | Montag Stiftung & Regionale2010, 2008 |
| **V. Installation of nature-based solutions (NBS) ("water-sensitive urban design")** | | | | | | | | | |
| GER/ SWE-1 | The aesthetic performance of urban landscape-based stormwater management systems | Different cities in Northern Europe (GER, SWE) | n/a | 2009 | n/a | Mixed | Other urban blue elements (A) | ✪ | Backhaus & Fryd, 2013 |
| NLD-2 | Rotterdam Water City 2035 | Rotterdam, NLD | XL | Ongoing since 2005 | n/a | n/a | Combination of different urban blue space types (N) | ✪ | van der Brugge & de Graaf, 2008 |
| UK-7 | Clever City London: Thamesmead | London, UK | GL | Ongoing since 2018 | n/a | n/a | Other urban blue elements (A) | n/a | CLEVER Cities, n.y. |
| ROM-2 | Clever City Sfântu Gheorghe | Sfântu Gheorghe, ROM | S | Ongoing since 2019 | n/a | n/a | Other urban blue elements (A) | n/a | CLEVER Cities, n.y. |

| | | | | | | | | | |
|---|---|---|---|---|---|---|---|---|---|
| **VI. Others (e.g. waterside estate action, holistic neighborhood upgrading programs)** | | | | | | | | | |
| GER-1 | Living by the water | Cologne, GER | XL | 2007 | 0.244 m. EUR (partial) | Public | Flowing inland waterbody (N) | ✪ | Montag Stiftung & Regionale2010, 2008 |
| FIN-1 | Assessing social impacts in urban waterfront regeneration | Helsinki, FIN | XL | 2000 | n/a | n/a | Large-scale waterbody connected to the ocean (N) | n/a | Sairinen & Kumpulainen, 2006 |
| GER-7 | New ways to the water | Essen, GER | XL | Ongoing since 2006 | n/a | Mixed | Combination of different urban blue space types (A, N) | ✪ | Essener Konsens, n.y. |
| PRT-1 | Urban Rehabilitation Program of Marinha de Silvalde | Espinho, PRT | XS | 2002 | 2.27 m. EUR | n/a | Combination of different urban blue space types (N) | ✪ | CCCB, 2018 |
| GRC-1 | Drapetsona-Keratsini | Drapetsona-Keratsini, GRC | S | 1999 | n/a | Public | Large-scale waterbody connected to the ocean (N) | ✪ | BBR & BMVBS, 2007 |
| GER-4 | The Socially Integrative City | Different cities in GER | n/a | Ongoing since 1999 | n/a | Public | Dependent on subproject; mostly flowing inland waterbodies (N) | n/a | DUH, 2017 |

[i] Classification modified from Dijkstra & Poelman (2012): Since the OECD-EC definition only considers towns with >50,000 inhabitants as small city, we added a category "XS" for cities with <50,000 inhabitants. GL = Global city. Projects in several cities are marked as n/a.

[ii] Classification after Völker et al. (2016). The nature of the waterbody is classified by (A) = artificial or (N) = natural.

[iii] ✪ = Projects that have been awarded or quoted as good practice examples of urban regeneration. The awards are mostly related to architecture and urban design (e.g. the European Price for Urban Public Space); only few honor any commitment to sustainability (e.g. awards for action on biodiversity or climate change) and other fields of action.

**(*PHI*)**: Public health intervention

## REFERENCES


The River Restoration Center (2009) The London Rivers Action Plan. A tool to help restore rivers for people and nature. Edited by The River Restoration Center. Bedford. Available online at: https://www.therrc.co.uk/lrap/lplan.pdf (accessed September 18, 2021).

Centre of Contemporary Culture of Barcelona (2021) The European Prize for Urban Public Space. A selection of the best works. Edited by Centre of Contemporary Culture of Barcelona (CCCB). Available online at: https://www.publicspace.org/en/works (accessed September 18, 2021).

Federal Ministry of the Interior, Building and Community (2020) Soziale Stadt. Edited by BMI. Available online at: https://www.staedtebaufoerderunginfo/DE/ProgrammeVor2020/SozialeStadt/Praxis/praxis_node.html (accessed September 18, 2021).

Project for Public Spaces (2008)Mixed use Dubai. Edited by Project for Public Spaces (PPS). Available online at: https://www.pps.org/article/mixed-use-indubai (accessed September 18, 2021).

Völker S, Matros J, Claßen T. Determining urban open spaces for health-related appropriations: a qualitative analysis on the significance of blue space. *Environ Earth Sci.* (2016). 75:1–18. doi: 10.1007/s12665-016-5839-3

Clever Cities (n.y.) Co-designing Locally tailored Ecological solutions for Value added, socially inclusive Regeneration Cities. Edited by Clever Cities. Available online at: https://clevercities.eu (accessed September 19, 2021).

Federal Ministry of Transport, Building and Urban Affairs (BMVBS) and Federal Office for Building and Regional Planning (2007) Strategies for upgrading the physical environment deprived urban areas. Examples of good practice Europe. Background study on the "Leipzig Charter on sustainable European cities) of the German EU council presidency. Edited by BMVBS and BBR. Berlin/Bonn (BBR-Online publication, 6). Available online at: https://www.bbsr.bund.de/BBSR/EN/publications/OnlinePublications/2007/ON062007.html (accessed September 18, 2021).

INHERIT (38). Inter-sectoral Health and Environment Research for Innovation. INHERIT database. Edited by INHERIT. Available online at: https://inherit.eu/ (accessed September 18, 2021).

Environmental Action Germany (2017) Grün. Sozial. Wertvoll. Gemeinsam Natur sozial benachteiligte Quartiere holen! Empfehlungen und Beispiele für Kommunen. Edited by DUH. Radolfzell. Available online at: https://www.duh.de/fileadmin/user_upload/download/Projektinformation/Kommunaler_Umweltschutz/Umweltgerechtigkeit/Gruenflaechen/Gruen.Sozial.Wertvoll._Gemeinsam-Natur-in-sozial-benachteiligte-Quartiere-holen.pdf (accessed September 18, 2021).

van der Brugge R,Graaf R. Transforming water infrastructure by linking water management and urban renewal Rotterdam. In*: First International Conference on Infrastructure Systems and Services: Building Networks for a Brighter Future (INFRA)*. Rotterdam, Netherlands, 10.11.2008 - 12.11.2008: IEEE, (2008). p. 1–7. doi: 10.1109/INFRA.2008.5439607

Sabarmati River Front Development Corporation (SRFDCL) and Ahmedabad Municipal Corporation (n.y.) Sabarmati Riverfront. Socializing a river and inclusive development. Available online at: https://www.niua.org/csc/assets/pdf/urban-planning/Sabarmati-Riverfront-Ahmedabad.pdf (accessed September 18, 2021).

Emschergenossenschaft (n.y.). Vielfältig. Lebendig. Attraktiv. Das Jahrhundertprojekt Emscher-Umbau - Neue Impulse für die Stadtentwicklung. Edited by Emschergenossenschaft. Available online at: https://www.eglv.de/app/uploads/2019/10/20120316_Broschu_re2_DE.pdf (accessed September 22, 2021).

Essener Konsens (n.y.). Von der Vision zur Wirklichkeit. Die wichtigsten Projekte zur Freiflächenentwicklung bis 2015. Edited by Essener Konsens. Available online at: http://www.neuewegezumwasser.de/download/Projektdoku_NWZW.pdf (accessed September 18, 2021).

Dijkstra L, Poelman H. (2012) Cities Europe: The new OECD-EC definition. Edited by European Commission (Regional Focus, 1). Available online at: https://ec.europa.eu/regional_policy/sources/docgener/focus/2012_01_city.pdf (accessed September 18, 2021).
